# Supplementary material for: Linear viscoelastic properties of the vertex model for epithelial tissues
Source: PLoS Comput Biol. 2022 May 19;18(5):e1010135. doi: 10.1371/journal.pcbi.1010135 (PMC9159552; doi:10.1371/journal.pcbi.1010135)
Supplement: S1 File — (PDF) [file pcbi.1010135.s001.pdf]

Linear Viscoelastic Properties of the Vertex Model for Epithelial Tissues

Sijie Tong, Navreeta K. Singh, Rastko Sknepnek, Andrej Košmrlj

Supporting Information

A The procedure for creating disordered tiling configurations

Fig. A shows the procedure to create disordered tiling configurations used to perform rheological simulations.

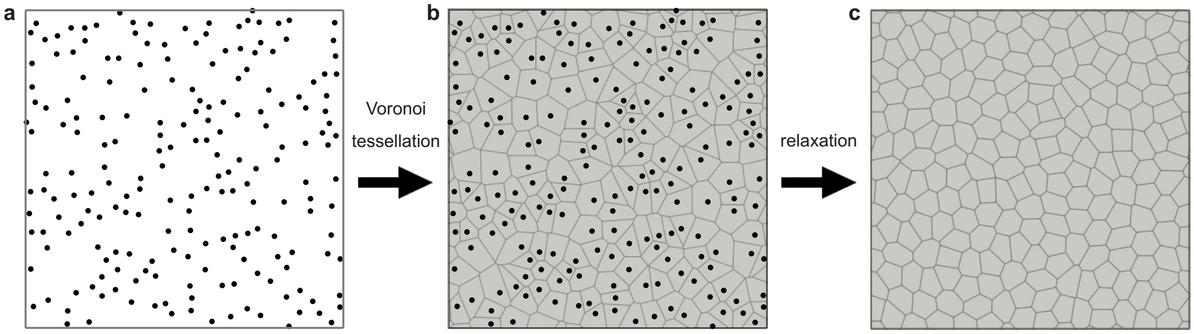

Fig A. The procedure of creating disordered tilings of polygons. (a) We first created a random point pattern of N non-overlapping points within a square box. (b) These points are used as seeds for Voronoi tessellation subject to periodic boundary condition. (c) The energy of the system was relaxed to a local energy minimum using the FIRE algorithm.

B Connection between stress response and rheology

Fig. B shows a typical average shear stress $\tau(t)$ in response to an applied oscillatory simple shear with the strain $\epsilon = \epsilon_0 \sin(\omega_0 t)$. The shear stress response can be represented as

$$\tau(t) = \tau_0 \sin(\omega_0 t + \delta) = \tau_0 \cos(\delta) \sin(\omega_0 t) + \tau_0 \sin(\delta) \cos(\omega_0 t).$$

The storage shear modulus is related to the in-phase response and is defined as $G' = (\tau_0/\epsilon_0) \cos\delta$. The loss shear modulus is related to the out-of-phase response and is defined as $G'' = (\tau_0/\epsilon_0) \sin\delta$ [1].

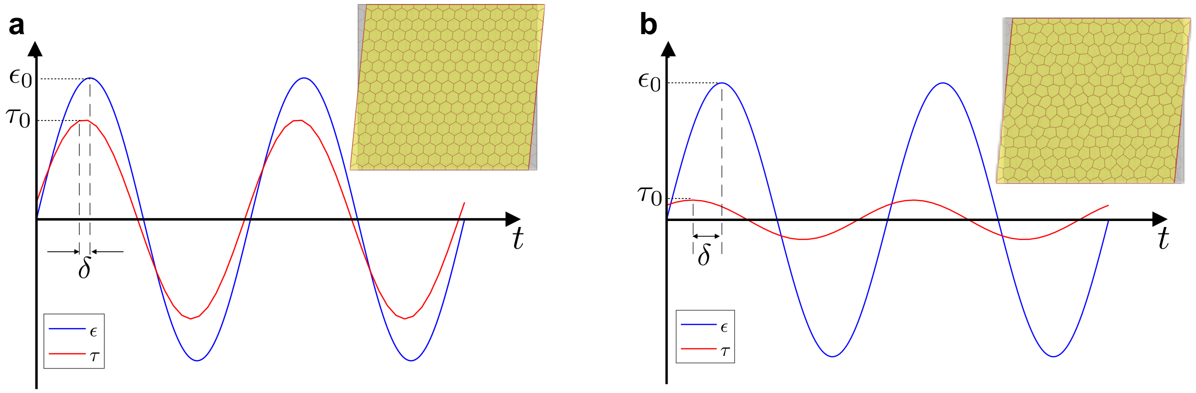

Fig B. Typical shear stress (red curve) as a function of time in response to a periodic shear strain (blue curve) in (a) the solid phase and (b) the fluid phase. The shear stress is averaged over all cells.

C Approach of the response stress towards the steady state

Here, we show an example of how the steady state shear stress $\tilde{\tau}(\omega_0)$ is measured in response to the applied oscillatory simple shear with a time period $T_0 = 27.7\gamma/(KA_0) = 2\pi/\omega_0$ for the shape parameter $p_0 = 3.723$ in hexagonal tiling, which is very close to the critical point $p_c \approx 3.722$ for the solid-fluid transition. The shear stress signal $\tau(t)$ was divided into blocks of length $T = 3T_0$, each containing 3 cycles of the time period of the driving shear deformation (see Fig. C). Within each block n, we performed the Fourier transform of $\tau(t)$ and obtained $\tilde{\tau}_n(\omega)$ as

$$\tilde{\tau}_n(\omega) = \frac{1}{T}\int_{(n-1)T}^{nT} \tau(t)e^{i\omega t}dt, \tag{S1}$$

where n is a positive integer. The value of $\tilde{\tau}_n(\omega_0)$ converges exponentially to the steady state value (see Fig. C), where the relaxation time is related to the characteristic timescales of the viscoelastic models (see Fig. 3c,d in the main text). For values of p_0 far away from p_c, the system quickly reaches a steady state (within 3–6 cycles). As p_0 approaches p_c the relaxation times become much longer, which is reflecting the diverging characteristic timescales of the viscoelastic models (see Fig. 3c,d in the main text).

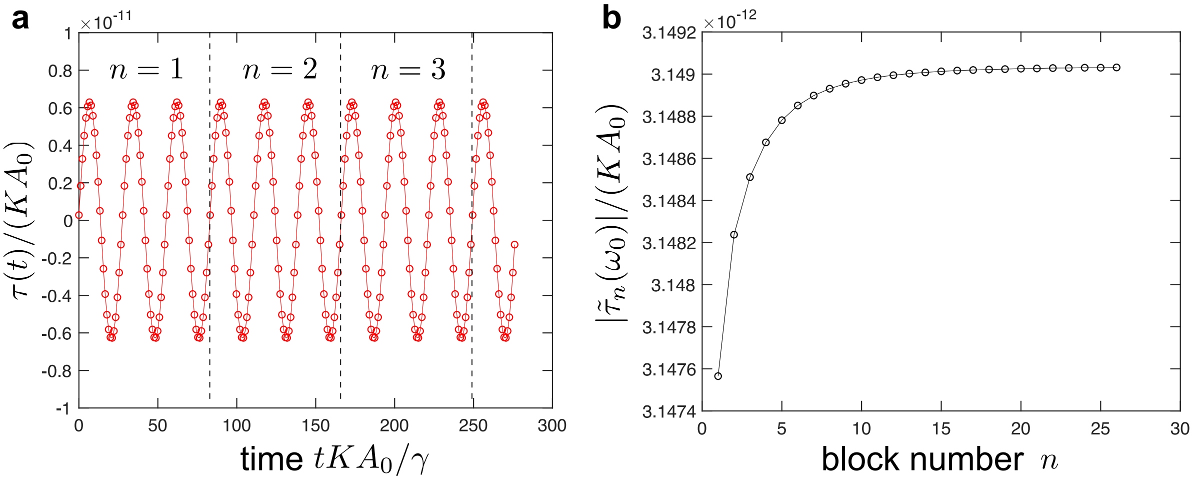

Fig C. Approach of the response shear stress towards the steady state. (a) The shear stress signal $\tau(t)$ was divided into blocks indicated by the vertical dashed lines. (b) Fourier transform of the response shear stress $\tilde{\tau}_n(\omega_0)$ at the driving frequency, ω_0, as a function of the block number, n.

D Effect of residual hydrostatic stress on the spring constants in the solid phase for hexagonal tilings

In the solid phase, we studied the rheology of the hexagonal tiling with each cell of area $A_C = A_0$ but with the perimeter P_C unequal to the preferred perimeter P_0, which induces residual hydrostatic stress in equilibrium. This residual stress can be eliminated if the lattice is uniformly rescaled by a factor α, which minimizes the following dimensionless energy per cell,

$$e_C(\alpha) = \frac{1}{2}\left(\alpha^2 - 1\right)^2 + \frac{\tilde{\Gamma}}{2}\left(\alpha p_C - p_0\right)^2, \tag{S2}$$

where $e_C = \frac{E_C}{KA_0^2}$, $\tilde{\Gamma} = \frac{\Gamma}{KA_0}$, $p_C = \frac{P_C}{\sqrt{A_0}} = \sqrt[4]{192} \approx 3.722$, i.e., α is the root of equation $e'_C(\alpha) = 0$. In the solid phase, $\alpha < 1$, and the system shrinks to relax the residual stress. At the solid-fluid transition point, $\alpha = 1$ since the area and perimeter of each cell match their preferred values simultaneously. If the residual stress is eliminated by rescaling the box, the rheology of the system subject to a simple shear can still be described by the SLS model, although the fitted values of spring constants are different, as shown in Fig. D.

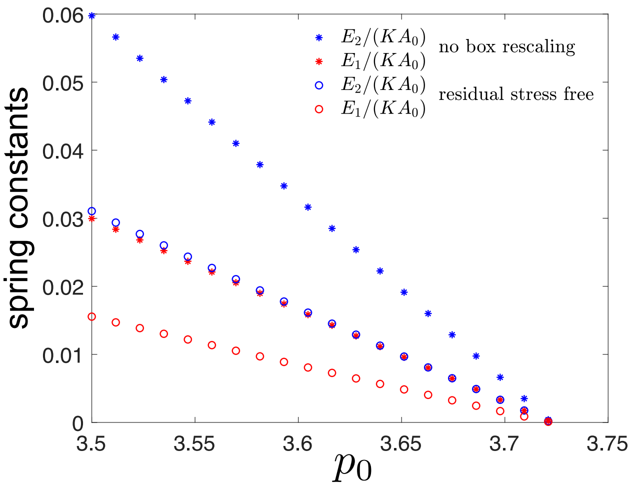

Fig D. The fitted spring constants in the solid phase for hexagonal tiling when the simulation box is not rescaled (closed symbols) and rescaled (open symbols) to eliminate residual stresses.

E Collapse of storage and loss shear moduli in the fluid phase for hexagonal tilings

In Fig. 2f in the main text, we showed the collapse of storage and loss shear moduli for the fluid phase for hexagonal tilings in the low frequency regime. Here we show the collapse in the high frequency range (see Fig. E), where we took into account that the relevant characteristic timescale scales as $\eta_2/E_2 \sim \gamma/(KA_0)$.

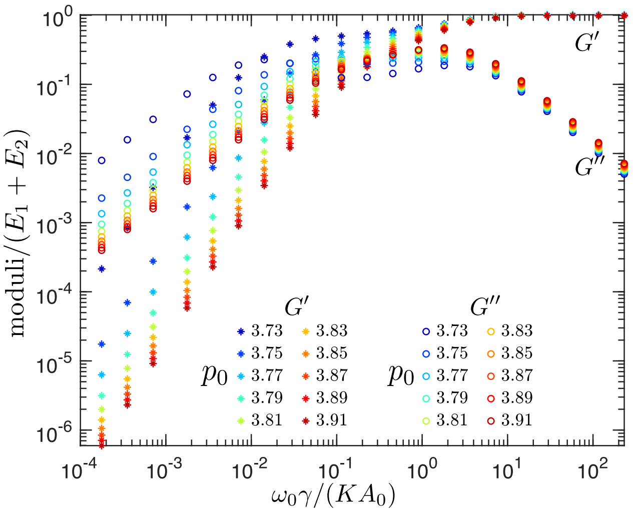

Fig E. The collapse of the storage (G') and loss (G'') shear moduli curves in the high frequency regime for different values of p_0 for the fluid phase.

F Effects of the initial perturbation of hexagonal tilings on the spring and dashpot constants in the fluid phase

We note that the rheological behavior in the fluid phase for hexagonal tilings is sensitive to the magnitude σ_D of the initial perturbation that was used to obtain different local energy minima configurations. In the main text, we showed the fitted values of spring and dashpot constants (Fig. 3) for the local energy minima configurations that were obtained by displacing each vertex coordinate of the hexagonal tiling by a Gaussian random variable with zero mean and standard deviation $\sigma_D = 1.5 \times 10^{-4}\sqrt{A_0}$. Here, we show that the fitted values of the spring and dashpot constants are somewhat sensitive to the magnitude σ_D of the random perturbation (see Fig. F).

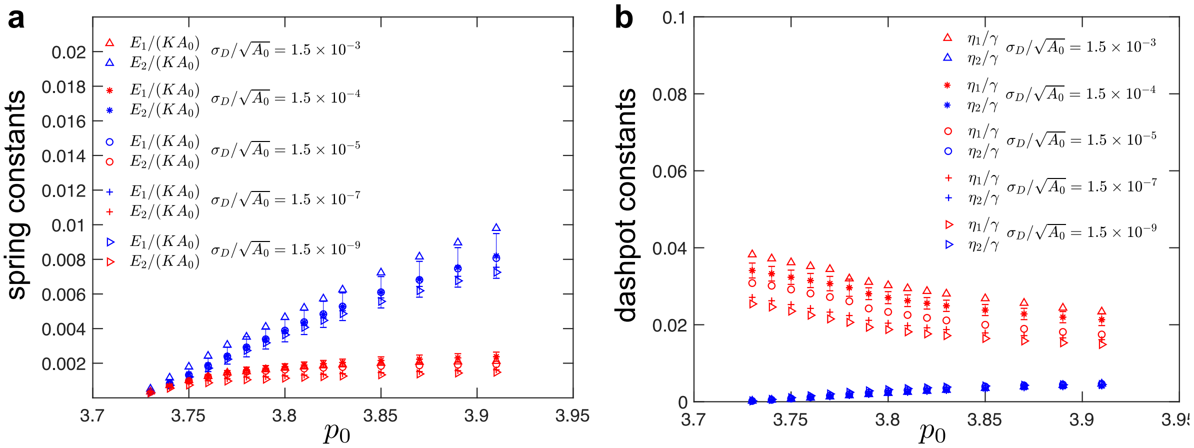

Fig F. Fitted values of (a) spring and (b) dashpot constants for hexagonal tilings under simple shear deformation as a function of the target cell-shape parameter, p_0, and the magnitude σ_D of the random perturbation that was used to obtain different local energy minima configurations in the fluid phase. Errorbars correspond to the standard deviation for simulations with $\sigma_D = 1.5 \times 10^{-4}\sqrt{A_0}$ that were repeated for configurations that correspond to different local energy minima.

G Tuning phase transition with different modes of pre-deformation.

In the main text, we showed that the solid-fluid transition point for hexagonal tilings can be tuned by uniaxially pre-compressing/stretching the system. Here, we discuss other pre-deformation modes that can also tune the transition.

We first derive the shear modulus due to affine deformation following a similar formalism as in Ref. [2]. There are two equivalent ways of derivation. The first one is to calculate the energy density of the system perturbed by an additional simple shear $\hat{\boldsymbol{F}} = \left(\begin{smallmatrix} 1 & \epsilon \\ 0 & 1 \end{smallmatrix}\right)$ where $\epsilon \ll 1$. For example, for the hexagonal tiling without any pre-deformation (i.e., regular hexagons), one can first calculate the vertex positions of a regular hexagon after being deformed by the affine transformation $\hat{\boldsymbol{F}}$. Then the perimeter of the deformed hexagon can be derived as

$$\frac{P}{6L_0} = 1 + \frac{3}{16}\epsilon^2 + o\left(\epsilon^4\right) \tag{S3}$$

where $L_0 = \sqrt{2A_0}/\sqrt{3\sqrt{3}}$ is the edge length of a regular hexagon before deformation. The area of the hexagon does not change after the simple shear deformation. With the knowledge of the perimeter and area of the deformed hexagon, one can calculate and expand the energy density from Eq. (1) in a power series in ϵ as

$$\frac{E}{NA_0} = \frac{1}{2} 3\sqrt{3}\Gamma \left(1 - \frac{p_0}{\sqrt{8\sqrt{3}}}\right) \epsilon^2 + o\left(\epsilon^4\right) \equiv \frac{1}{2} G_{\text{affine}} \epsilon^2 + o\left(\epsilon^4\right), \tag{S4}$$

where we omitted the constant term. The quadratic term characterizes the linear response of the system, which gives the shear modulus as in Eq. (7) in the main text. The second approach is to directly use the expression for the stress tensor Eq. (3). After obtaining the vertex positions of a hexagon perturbed by a simple shear $\hat{\boldsymbol{F}}$, one can calculate the shear stress and expand in a power series as $\tau = \hat{\sigma}_{xy} = G_{\text{affine}}\epsilon + o\left(\epsilon^2\right)$. The coefficient of the leading order term in ϵ is the shear modulus, which coincides with the modulus from the energy calculation. Similar derivation of the shear modulus can be carried out for the pre-deformed hexagonal tilings.

If the hexagonal tiling is pre-deformed biaxially according to the deformation gradient $\hat{\boldsymbol{F}} = \left(\begin{smallmatrix} a & 0 \\ 0 & a \end{smallmatrix}\right)$, then the shear modulus due to the affine deformation becomes

$$G_{\text{affine}} = 3\sqrt{3}\Gamma \left(1 - \frac{p_0}{a\sqrt{8\sqrt{3}}}\right). \tag{S5}$$

By setting G_{affine} to 0, the phase boundary in the $a - p_0$ plane is

$$p_c(a) = a\sqrt{8\sqrt{3}}. \tag{S6}$$

Similarly, consider a pure shear pre-deformation described by the deformation gradient $\hat{\boldsymbol{F}} = \left(\begin{smallmatrix} a & 0 \\ 0 & 1/a \end{smallmatrix}\right)$. The shear modulus due to the affine deformation then becomes

$$G_{\text{affine}} = \frac{2\sqrt{2}\left(1 + \sqrt{1+3a^4} + 3a^4\sqrt{1+3a^4}\right)\left(2\sqrt{2}\; 3^{1/4}(1 + \sqrt{1+3a^4}) - 3ap_0\right)\Gamma}{3^{7/4}a(1+3a^4)^{3/2}}, \tag{S7}$$

and the phase boundary is

$$p_c(a) = \sqrt{8\sqrt{3}}\; \frac{(1 + \sqrt{1+3a^4})}{3a}. \tag{S8}$$

The phase diagrams for a hexagonal tiling that is under biaxial or pure shear pre-deformation are shown in Fig. G. The phase boundary in the $a - p_0$ plane follows Eq. (S6) for biaxial pre-deformation and Eq. (S8) for pure shear pre-deformation. The system can be rigidified by stretching or shearing.

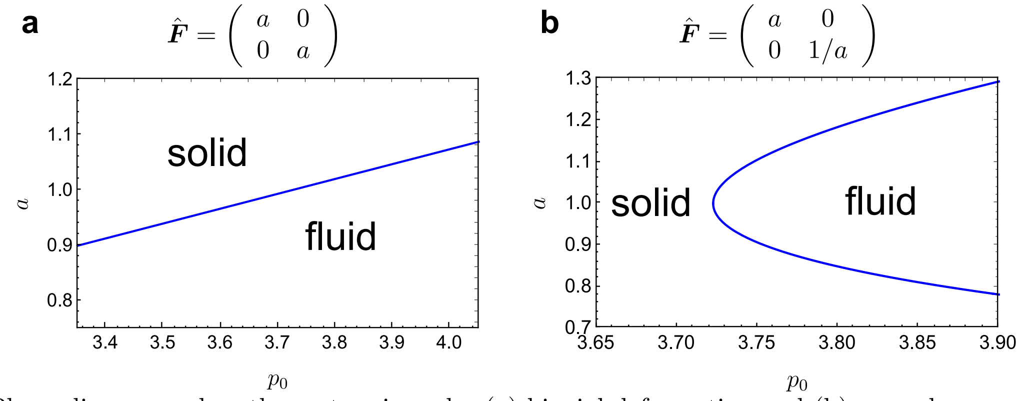

Fig G. Phase diagrams when the system is under (a) biaxial deformation and (b) pure shear.

H Spectrum of the normal modes for hexagonal tilings

We calculated the eigenvalues λ of the Hessian matrix $\frac{\partial^2 E}{\partial \mathbf{r}_i \partial \mathbf{r}_j}$ associated with the energy functional of the vertex model for hexagonal tiling. We associate each positive eigenvalue λ with a corresponding eigenfrequency $\omega = \sqrt{\lambda}$, which describes the oscillations of that mode as the system is perturbed about its stable point. Fig. H shows the cumulative density of states, which is defined as [3]

$$N(\omega) = \int_{0^+}^{\infty} D(\omega')d\omega' + N(\lambda = 0)\theta(\omega), \tag{S9}$$

where $D(\omega)$ is density of states, $N(\lambda = 0)$ is the fraction of zero eigenvalues and $\theta(\omega)$ is the Heaviside step function. In the solid phase, there are no zero modes other than the two translational rigid body motions. In the fluid phase, however, approximately half of the eigenmodes are zero modes. As p_0 approaches the critical value p_c in both solid and fluid phase, $N(\omega)$ curves move to the left so the system becomes softer, which is consistent with the dependence of the spring constants on p_0 shown in Fig. 3a in the main text.

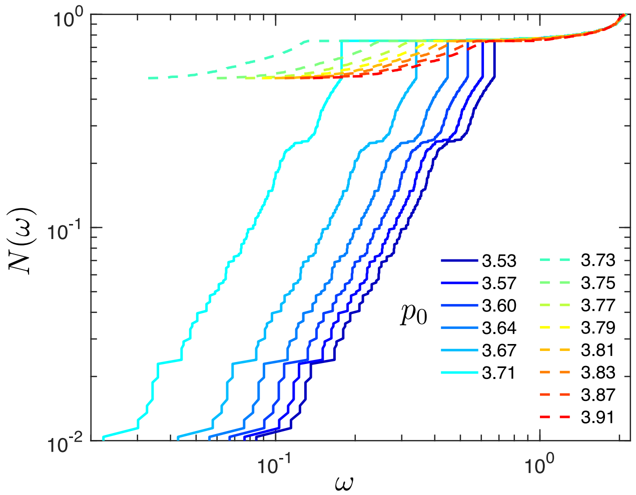

Fig H. Cumulative density of states in the solid phase (solid lines) and in the fluid phase (dashed lines) for hexagonal tilings.

I Raw data of storage and loss shear moduli for disordered tilings

Fig. I shows the raw data of storage and loss shear moduli for disordered tilings for a range of values of p_0. Each color represents the storage and loss shear moduli for one disordered tiling configuration. These data are used to calculate the average storage and loss shear moduli for each value of p_0. From the raw data one can see large variability in storage and loss moduli when p_0 is close to the critical value of the solid-fluid transition. When $p_0 = 3.93$ and $p_0 = 3.95$, there is a mixture of solid and fluid configurations since some storage moduli plateau at a nonzero constant value and some vanish in the low frequency limit.

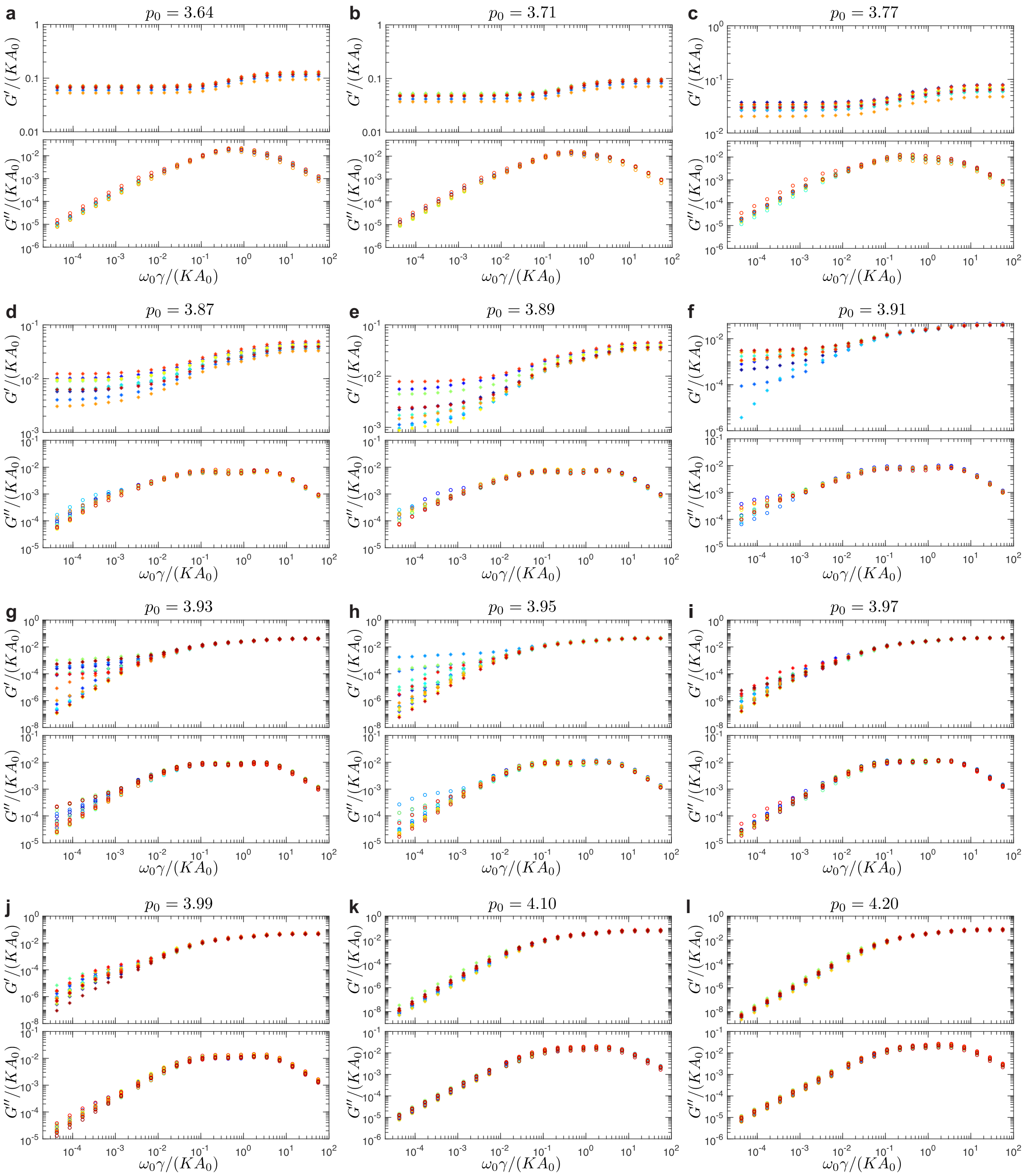

Fig I. Raw data of storage (G') and loss (G'') shear moduli for disordered tilings. The variability in storage and loss moduli increases as p_0 approaches the critical value of the solid-fluid transition.

J Raw data of storage and loss bulk moduli for disordered tilings

Fig. J shows the raw data of storage and loss bulk moduli for disordered tilings at a few representative values of p_0. The storage and loss moduli have high variability when p_0 is close to the critical value of solid-fluid transition ($p_0 = 3.93$).

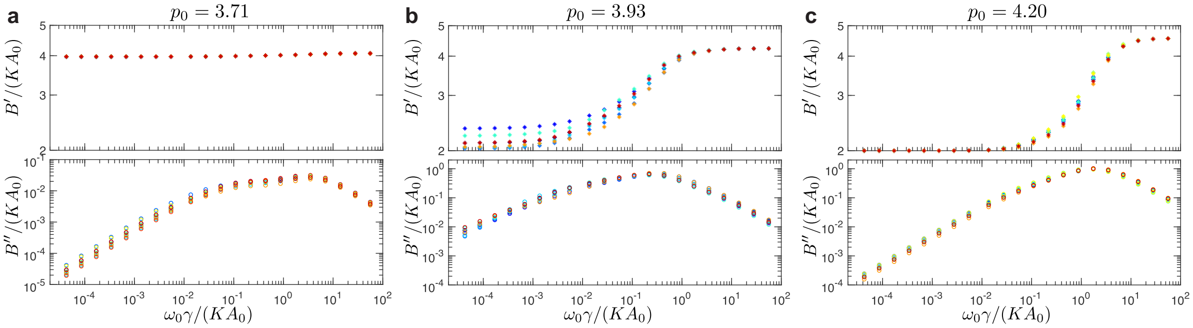

Fig J. Raw data of storage (B') and loss (B'') bulk moduli for disordered tilings.

K Comparison of fits of shear moduli based on different spring-dashpot models for disordered tilings

Fig. K shows the fits of average storage and loss shear moduli based on different spring-dashpot models for disordered tilings at $p_0 = 3.71$. Adding more Maxwell elements in parallel to the Standard Linear Solid model increases the accuracy of fits. In Fig. Kc with the most accurate fit presented here, however, the fitted curve of loss modulus goes up and down through the simulation curve. This manifests the characteristic of fit with high order polynomials and indicates that addition of more Maxwell elements does not fully capture the behavior of the shear moduli obtained from the simulations.

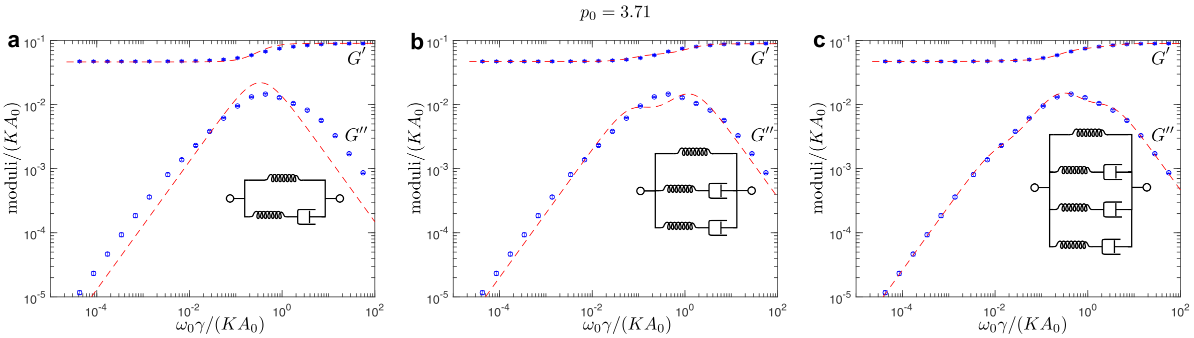

Fig K. Fits of average shear moduli based on different spring-dashpot models for disordered tilings at $p_0 = 3.71$. Red dashed lines are the fits. Blue dots represent the moduli data obtained from the simulations. (a) shows the fits based on the Standard Linear Solid (SLS) model. The fits in (b) and (c) are based on spring-dashpot models with additional Maxwell elements in parallel to the SLS model. The insets of each plot show the representation of the corresponding spring-dashpot models.

L System size effect for disordered tilings

Fig. L shows the storage and loss shear moduli for disordered tilings of different sizes at $p_0 = 3.71$. The system sizes have no effect at high frequency of shearing. At intermediate frequency, the loss modulus has an anomalous scaling exponent, i.e., $\sim \omega_0^\alpha$ with $\alpha \approx 0.73$, which changes from being linear in low frequency. This crossover moves to lower frequencies as the system size increases.

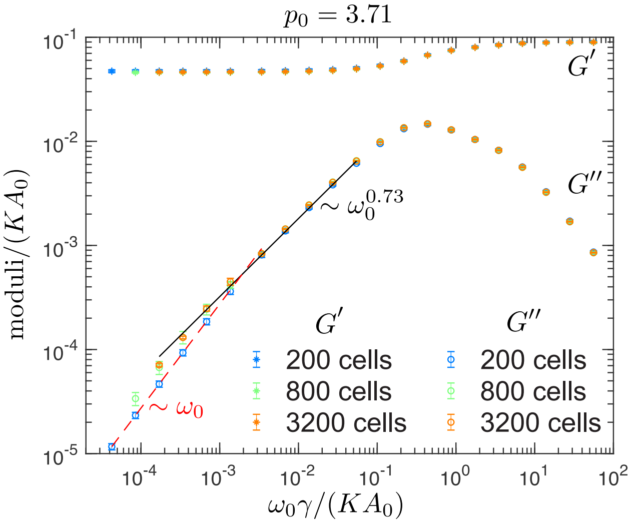

Fig L. Storage and loss shear moduli for disordered tilings of different system sizes at $p_0 = 3.71$.

References

1. Larson RG. The Structure and Rheology of Complex Fluids. vol. 150. Oxford University Press New York; 1999.

2. Staple D, Farhadifar R, Röper JC, Aigouy B, Eaton S, Jülicher F. Mechanics and remodelling of cell packings in epithelia. The European Physical Journal E. 2010;33(2):117–127.

3. Bi D, Lopez J, Schwarz JM, Manning ML. A density-independent rigidity transition in biological tissues. Nature Physics. 2015;11(12):1074–1079.
