# Supplementary material for: Linear viscoelastic properties of the vertex model for epithelial tissues
Source: PLoS Comput Biol. 2022 May 19;18(5):e1010135. doi: 10.1371/journal.pcbi.1010135 (PMC9159552; doi:10.1371/journal.pcbi.1010135)
Supplement: Attachment — Submitted filename: response_letter.pdf [file pcbi.1010135.s002.pdf]

Dear Dr. Bollenbach and Dr. Haugh,

Thank you for obtaining reviews for our manuscript PCOMPBIOL-D-22-00136 titled "Linear viscoelastic properties of the vertex model for epithelial tissues". Both reviewers were positive but they also provided some constructive comments, which helped us clarify simulation details and improve the discussion of our results. Below we describe in detail how every reviewer comment (black text) was addressed (blue text) along with the specific revisions we made to the manuscript, which are highlighted in blue color in the revised manuscript. Furthermore, we have made the simulation and analysis codes publicly available on GitHub (`https://github.com/sknepneklab/RheoVM`).

We hope that the revised and improved manuscript is now suitable for publication in PLOS Computational Biology.

Sincerely,

Andrej Košmrlj on behalf of all the authors

Response to Reviewer 1:

The manuscript "Linear viscoelastic properties of the vertex model for epithelial tissues" by Tong et al. explores the rheological properties of epithelial tissue in computer simulations using the vertex model. In contrast to previous models which were considering mechanical properties of the vertex model on long time scales only, this analysis now provides insight into viscoelastic mechanical properties at a large range of time scales.

The analysis is presented for ordered and disordered hexagonal tilings of the epithelium in the solid-like and fluid-like regime as tuned by the shape parameter $p_0 = P_0/\sqrt{A_0}$. Here, P_0 is the preferred cellular perimeter and A_0 is the preferred cellular surface area. Furthermore, the authors report the rheological properties for disordered polygonal tilings at different values of the parameter p_0. For both cases, regular hexagonal and irregular polygonal tilings, moduli with regards to shear and bulk deformations are reported. Interestingly, the authors find that for most parameter regimes, the mechanics of the epithelium can be captured by established viscoelastic models such as the Standard Linear Solid (SLS) and the Burgers model, which can describe the full time-scale dependent mechanics of the material with three or four material constants (two elastic constants and one or two viscosities), respectively. The authors analyse how these effective material constants depend on the shape parameter p_0, the nature of the deformation (shear or bulk) and the initial configuration of the epithelium (regular hexagonal or irregular polygonal). Within their approach the authors consider only friction between cells and the substrate and neglect internal dissipation within the tissue. My impression is that the research has been carried out with high technical rigor and that the paper is well written and thought through. Furthermore, I can see that the work makes a substantial new contribution to the field.

Therefore, I recommend to publish the paper in PLOS Computational Biology after revision with regards to the few queries that I listed below.

Reply: We thank the reviewer for the kind remarks and for the constructive comments, which helped us improve the manuscript.

Major:

The authors state that deformations have been applied in their study at a small amplitude of $\varepsilon_0 = 10^{-7}$ to ensure that the mechanical response is in the linear regime. Clearly, that order of magnitude of deformation is not of any practical relevance in reality. Therefore, can the authors clarify, to which extent their analysis changes if deformations are stepped up to higher values such as the 1-10% range?

Reply: The analysis presented in this study is indeed limited to the linear response regime. Large deformations that are, as the reviewer pointed out, of significant relevance for real tissues are beyond the scope of this work. Large deformations lead to several non-trivial effects. For example, Ref. [44] (Huang J, et al., arXiv:210910374) showed that large quasistatic shearing strain first solidifies the model tissue, which then yields via plastic T1 events as the shear strain is increased. As a result, the stress-strain curves are highly non-linear. This makes the rheological analysis over the full range of driving frequencies challenging and at present poorly understood. Therefore, the main focus of this study was to map out the linear response of the vertex model in the full dynamic range and hence provide the baseline for its applications in studies of real tissues. Note that our linear rheological analysis of uniaxially predeformed tissues with strains up to 5% (see Fig. 10) can be understood as the tangent moduli to the general stress-strain curves.

We have added the following paragraph to the 'Discussion and conclusions' section:

"It is also important to note that our work focuses on the rheological behavior in the tangent moduli approximation to a general stress-strain curves, where applied shear and bulk deformations are infinitesimal with respect to a potentially significantly predeformed state (e.g., see Fig. 10). This is clearly an idealized case and, in reality, one would be interested in the response to higher values of the applied deformation ($\gtrsim 1 - 10\%$). It would be possible to study such finite deformations using our simulation protocol. Interpreting the results would, however, be more challenging, e.g., due to shear-driven rigidity transition in model tissues and the presence of plastic events [44,46]."

Minor:

- Figure 4 would benefit from an additional panel where an exemplary irregular polygonal lattice is shown.

Reply: Configurations used in simulation included in Fig. 4 are very similar to the one that is already shown in Fig. 1b. Instead of adding an additional column to Fig. 4, we now refer the readers to Fig. 1b in the main text as well as in the captions of Fig. 4, when discussing simulation results for disordered tilings.

- Section 7 of the Supplement would profit from a more detailed explanation or referencing to account for the derived equations.

Reply: Following the reviewer's suggestion, we have reordered the presentation in Section G (Section S7 in the original document) and provided additional information about how the effective shear moduli are calculated.

Response to Reviewer 2

The manuscript by Tong. et al. carefully explores the viscoelastic properties of the Vertex model- a basic model that has been extensively used to study biophysical properties of epithelial tissues. The authors analyze the response of the cellular network under small shear and bulk deformations for both a regular hexagonal network and irregular topological configurations. They found that standard spring-dashpot models quantitatively explain the complex behaviors of the Vertex model under these deformations. I found the method, analysis, and results fascinating and recommend this paper for publication.

Reply: We thank the reviewer for the insightful remarks and for the constructive comments, which helped us improve the manuscript by providing additional simulation details and additional discussion of our results.

However, I recommend the authors elaborate on a few details:

1- It is not clear how the periodic boundary conditions are implemented: First, how do they treat the PBC to ensure no external stress on cells? How is the PBC implemented for simple shear simulations? The correct implementation of PBC for simple shear simulations requires careful movement of the vertices in each image boundary.

Reply: We thank the reviewer for alerting us that we didn't clearly specify how periodic boundary conditions were treated in our simulations. For a vertex i with a position vector $\mathbf{r}_i(t)$ we also consider its periodic images at positions $\mathbf{r}_i(t) + m_1\mathbf{a}_1(t) + m_2\mathbf{a}_2(t)$, where $\mathbf{a}_{1,2}(t)$ are the unit cell vectors of the periodic box and $m_{1,2}$ are integers. For cells that cross the periodic boundary, junction lengths are computed using the minimum image convention. The affine deformation $\hat{\boldsymbol{F}}(t)$ was applied to all vertices inside the periodic box as well as to the periodic images. Note that the unit cell vectors of the periodic box transform as $\mathbf{a}_{1,2}(t) \to \hat{\boldsymbol{F}}(t)\mathbf{a}_{1,2}(0)$ due to the affine deformation.

We have added the following paragraph to the 'Dynamics and probing the rheology' section:

"We applied an oscillatory affine deformation to investigate the rheological behavior of the vertex model. The affine deformation can be described by a deformation gradient tensor defined as $\hat{\boldsymbol{F}} = \partial\mathbf{x}/\partial\mathbf{X}_0$, where the mapping $\mathbf{x} = \mathbf{x}\left(\mathbf{X}_0, t\right)$ maps the reference configuration $\mathbf{X}_0$ to a spatial configuration $\mathbf{x}$ at time t. Note that the affine deformation was applied to all vertices inside the bounding box as well as to the periodic images of vertices, which ensured deformation of cells across periodic boundaries. Specifically, for a vertex i with a position vector $\mathbf{r}_i(t)$ in a deformed configuration, we also consider its periodic images at positions $\mathbf{r}_i(t) + m_1\mathbf{a}_1(t) + m_2\mathbf{a}_2(t)$, where $\mathbf{a}_{1,2}(t)$ are the unit cell vectors of the periodic box and $m_{1,2}$ are integers. For cells that cross the periodic boundary, junction lengths are computed using the minimum image convention. Note that the unit cell vectors of the periodic box transform as $\mathbf{a}_{1,2}(t) = \hat{\boldsymbol{F}}(t)\mathbf{a}_{1,2}(0)$ due to the affine deformation."

The reviewer is correct that the periodic boundaries in general impose additional stresses on the cells. As we mentioned in the 'Simulation setup' section, the preferred areas of cells were chosen such that the total area of the simulation box, L^2, matches the sum of the preferred areas of all cells in the box. For regular hexagonal tilings, the cell areas A_C were set to equal to the preferred values A_0. However, in the solid phase there is in general still some residual hydrostatic stress due to the mismatch of the cell perimeter P_C from their preferred values P_0. This hydrostatic stress could be eliminated by appropriate rescaling the size of the simulation box. This hydrostatic stress, however, does not qualitatively affect the rheological behavior of the system (see Sec. D in S1 File

for further discussion).

2- According to [50], the ground state transition from solid hexagonal network to liquid irregular network happens in multiple steps: First transition line occurs at a state that cells are free to change their shape, but the topology should be hexagonal. After the second transition line, cells can freely change their shapes, but only 5, 6, and 7 sided cells are allowed, and so on. Consequently, the shear modulus vanishes in multiple transitions, and a fully disordered network will have zero shear modulus only for p

Reply: We agree with the reviewer about findings in Ref. [50] (Staple, et al., EPJE 33, 117 (2010)). The reviewer comment, however, seems to be cut mid-sentence. We comment that Bi, et al. (Ref. [51]), showed that for $p_0 > p_c$, where p_c is a critical value, the energy barrier for cell neighbor exchanges vanishes. For a hexagonal tiling, $p_c \approx 3.722$, while for a disordered tiling $p_c \approx 3.92$. At that point, a large number of zero-energy modes appears in the spectrum and the system becomes soft with a vanishing shear modulus in the quasistatic limit. Note that the value of the quasistatic shear modulus remains zero as the value of p_0 is further increased, which is in agreement with the Section 4 in Ref. [50]. Thus the shear modulus does not vanish in multiple transitions.

3- Cell size variation dramatically increases for a larger value of Γ_C. Have the authors done simulations to measure tissue rheology for different values of Γ_C?

Reply: In the dimensionless form, the vertex model depends on two parameters p_0 and $\Gamma_C/(\kappa_C A_0)$. In this study, we used the ratio $\Gamma_C/(\kappa_C A_0) \approx 0.298$, since this ratio does not qualitatively change the behavior of the vertex model as was shown in Refs. [20,41]. The analysis of the rheological response for different values of this ratio is given in Tong, et al., arXiv:2202.03261 (also ref. [76] in the revised manuscript), where we showed that the perimeter stiffness Γ and the area stiffness K affects the low-frequency and high-frequency rheological behavior, respectively. To make this point clear, we added the following paragraph to the 'Discussion and conclusions' section of the revised manuscript:

"In this work we kept the ratio $\Gamma/(KA_0)$ fixed, since this ratio does not qualitatively change the behavior of the vertex model [20,41]. In Ref. [76], however, we further explored the effect of parameter $\Gamma/(KA_0)$, where we showed that the perimeter stiffness Γ and the area stiffness K affects the low-frequency and high-frequency rheological behavior, respectively."

4- Cells in many tissues have anisotropic mechanical properties. For example, in a fly embryo or wing, cells are polarized, and cellular polarizations are mostly aligned (all cells have the same polarization orientation). Proteins that determine the polarization of cells also affect cell mechanics such that some boundaries are under higher tension than others. Can the authors comment on how tissue polarization might affect tissue rheology?

Reply: The reviewer raises a very interesting question. Cell polarisation plays an important role in epithelia especially in embryos. The vertex model in its basic form, as used in this study, does not include such effect. While it is possible to extend the vertex model to include cell polarity either by imposing anisotropy "by hand" or by coupling mechanical degrees of freedom to the kinetics of molecular motors that produce active tension, this is beyond the scope of the present study. We, however, comment that there is nothing that would prevent applying present formalism

to analyse such models. We have added the following paragraph to ‘Discussion and conclusions’ section in the revised manuscript:

“The vertex model provides a rather coarse description of real epithelial tissues and omits many important aspects such as cell polarization, chemical signalling, cells’ ability to actively adjust their properties in response to their environment, etc. The framework presented in this study would, however, be able to address the linear response in the presence of such effects, provided that the vertex model is suitably augmented [83-85].”
